# Modulation of plant growth *in vivo* and identification of kinase substrates using an analog-sensitive variant of CYCLIN-DEPENDENT KINASE A;1

**DOI:** 10.1186/s12870-016-0900-7

**Published:** 2016-09-26

**Authors:** Hirofumi Harashima, Nico Dissmeyer, Philippe Hammann, Yuko Nomura, Katharina Kramer, Hirofumi Nakagami, Arp Schnittger

**Affiliations:** 1 Department of Molecular Mechanisms of Phenotypic Plasticity, Institut de Biologie Moléculaire des Plantes du CNRS, IBMP-CNRS - UPR2357, Université de Strasbourg, F-67084 Strasbourg, France; 2 Trinationales Institut für Pflanzenforschung, F-67084 Strasbourg Cedex, France; 3 Present address: RIKEN Center for Sustainable Resource Science, 1-7-22 Suehiro, Tsurumi, Yokohama, Kanagawa 230-0045 Japan; 4 Present address: Leibniz Institute of Plant Biochemistry (IPB), Independent Junior Research Group on Protein Recognition and Degradation, Weinberg 3, D-06120 Halle, (Saale) Germany; 5 Plateforme protéomique Strasbourg Esplanade, Institut de Biologie Moléculaire et Cellulaire FRC1589-CNRS, F-67084 Strasbourg, France; 6 Plant Proteomics Research Unit, RIKEN Center for Sustainable Resource Science, 1-7-22 Suehiro-cho, Tsurumi Yokohama, 230-0045 Japan; 7 Max Planck Institute for Plant Breeding Research, Basic Immune System of Plants / Protein Mass Spectrometry, Carl-von-Linne-Weg 10, 50829 Cologne, Germany; 8 Department of Developmental Biology, University of Hamburg, Biozentrum Klein Flottbek, Ohnhorststr. 18, D-22609 Hamburg, Germany

**Keywords:** Kinase, Substrate, Phosphorylation, Cell cycle, Mitosis, *Arabidopsis*

## Abstract

**Background:**

Modulation of protein activity by phosphorylation through kinases and subsequent de-phosphorylation by phosphatases is one of the most prominent cellular control mechanisms. Thus, identification of kinase substrates is pivotal for the understanding of many – if not all – molecular biological processes. Equally, the possibility to deliberately tune kinase activity is of great value to analyze the biological process controlled by a particular kinase.

**Results:**

Here we have applied a chemical genetic approach and generated an analog-sensitive version of CDKA;1, the central cell-cycle regulator in *Arabidopsis* and homolog of the yeast Cdc2/CDC28 kinases. This variant could largely rescue a *cdka;1* mutant and is biochemically active, albeit less than the wild type. Applying bulky kinase inhibitors allowed the reduction of kinase activity in an organismic context in vivo and the modulation of plant growth. To isolate CDK substrates, we have adopted a two-dimensional differential gel electrophoresis strategy, and searched for proteins that showed mobility changes in fluorescently labeled extracts from plants expressing the analog-sensitive version of CDKA;1 with and without adding a bulky ATP variant. A pilot set of five proteins involved in a range of different processes could be confirmed in independent kinase assays to be phosphorylated by CDKA;1 approving the applicability of the here-developed method to identify substrates.

**Conclusion:**

The here presented generation of an analog-sensitive CDKA;1 version is functional and represent a novel tool to modulate kinase activity in vivo and identify kinase substrates. Our here performed pilot screen led to the identification of CDK targets that link cell proliferation control to sugar metabolism, proline proteolysis, and glucosinolate production providing a hint how cell proliferation and growth are integrated with plant development and physiology.

**Electronic supplementary material:**

The online version of this article (doi:10.1186/s12870-016-0900-7) contains supplementary material, which is available to authorized users.

## Background

Almost every aspect of cellular life relies on the dynamic addition and removal of phosphate groups on target proteins. Consequently, nearly 5 % of all genes of the model plant *Arabidopsis thaliana* were found to encode for protein kinases and protein phosphatases [1–4]. A paradigm for the importance of phospho-control is the regulation of the eukaryotic cell cycle. Progression through the cell cycle is controlled by heterodimeric enzymes comprised of a kinase subunit, called cyclin-dependent kinase (CDK), and a cyclin regulatory subunit [5]. Substantial work in yeast and animal model systems has shown that high kinase activity levels are in particular required to promote the transition from a gap phase (G1) into S phase where the nuclear DNA becomes replicated and from a second gap phase (G2) into M phase (mitosis) during which the chromosomes are distributed to the newly forming daughter cells. At these two major control points, CDK-cyclin complexes phosphorylate a plethora of target proteins. For instance in budding yeast, more than 300 proteins have been found to be substrates of CDC28 representing approximately 5 % of its proteome [6, 7]. Interestingly, some CDK substrates act outside of the core cell cycle connecting cell proliferation with cell differentiation, energy metabolism or other physiological processes such as redox regulation [8–10]. However, currently very little is known about the molecular basis of the integration of the cell cycle with other cell-physiological processes.

The homolog of the yeast *Cdc2/CDC28* gene is the *Arabidopsis CDKA;1*, which is the only *Arabidopsis* CDK that contains the conserved PSTAIRE cyclin-binding motif also found in animal Cdk1, Cdk2 and Cdk3 proteins. Moreover, CDKA;1 - in contrast to other plant-specific cell-cycle related CDKs - can complement the fission yeast *cdc2* and the budding yeast *cdc28* mutants [11–13]. CDKA;1 expression is linked to proliferation competence and has a key function in controlling S-phase entry next to a role in mitosis hence combining aspects of animal Cdk1 and Cdk2 kinases [14, 15]. This finding also raises the question to what degree CDKA;1 and Cdk1-type kinases from other organisms operate on homologous substrates in conserved pathways and what plant-specific CDK substrates are.

The detection of potentially plant-specific CDK targets is also key to understand how the cell cycle is integrated into plant development and growth [16], especially in the light of plants being the major source of food and feed for mankind and livestock, respectively and the prospect of plants as alternative resources of energy and raw materials. However, the identification of targets of specific protein kinases is a challenging task due to the high degree of structural and mechanistic conservation of the catalytic cores of all protein kinases and so far only very few substrates for plant cell-cycle kinases have been identified in an unbiased manner, i.e. not by comparison with substrates from other species [10, 17].

**Fig. 1:**
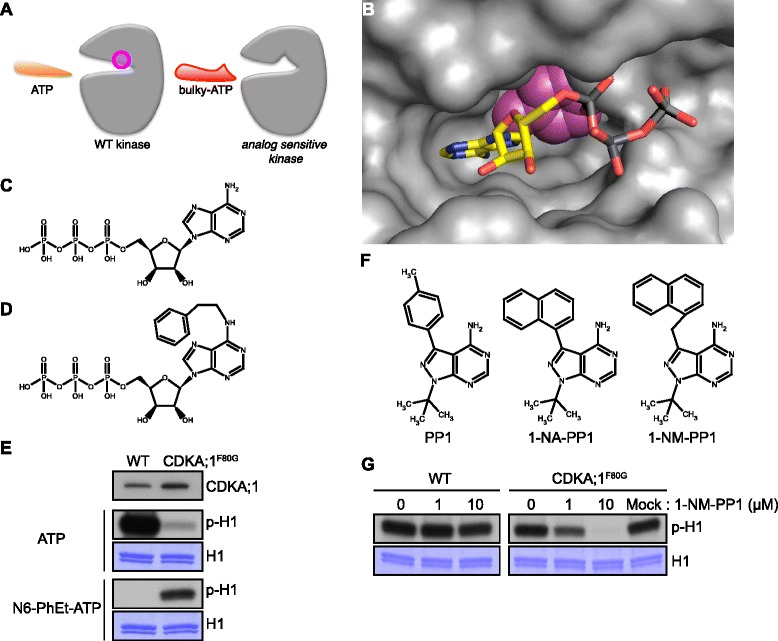
Generation and characterization of an analog-sensitive variant of CDKA;1. **a** Sketch of an analog-sensitive kinase variant (*right*) that has an enlarged ATP-binding pocket in comparison with the wild-type version (*left*) through exchanging a ‘gatekeeper’ amino acid (position in magenta), typically a large amino acid, in the wild-type version with a small one such as Gly. An analog-sensitive kinase can use regular ATP but also bulky derivatives that cannot be used by the wild-type variant as a phosphate donor (see also below). **b** Computed 3D structure of the ATP binding pocket of CDKA;1. In magenta, the space occupied at the bottom of the pocket by the gatekeeper amino acid Phenylalanine (Phe/F) 80 in the wild-type kinase that will be enlarged by the F80 to Glycine (Gly/G) mutation. **c** Structure of adenosine triphosphate (ATP). **d** Structure of the bulky-ATP derivate N^6^-(2-Phenylethyl)adenosine-5′-O-triphosphate (N6-PhEt-ATP). **e** In vitro kinase assay with wild-type and the analog-sensitive CDKA;1 (CDKA;1^F80G^) kinases using CYCD3;1 as a cyclin partner and histone H1 as a generic substrate. First lane from the top, protein blotting reveals equal amounts of CDKA;1 proteins in the reaction. Second lane, kinase assays with [γ-^32^P]-ATP as a phosphate donor. Forth lane, kinase assays with [γ-^32^P]-N6-PhEt-ATP as a phosphate donor. Proteins were subjected to SDS-PAGE after the kinase reaction and stained with Coomassie brilliant blue R-250 demonstrating equal loading of the substrate, lane three and five from the top. Abbreviations: p-H1 for radio-labeled histone H1 resulting from kinase assays with radio-labeled ATP, H1 for histone H1. **f** Structure of the broad band kinase inhibitor 4-amino-1-*tert*-butyl-3-phenylpyrazolo[3,4-*d*]pyrimidine (PP1) on the left and the bulky analogs 4-amino-1-*tert*-butyl-3-(1′-naphthyl)pyrazolo[3,4-*d*]pyrimidine (1-NA-PP1) in the middle as well as 4-amino-1-*tert*-butyl-3-(1′-naphthylmethyl)pyrazolo[3,4-*d*]pyrimidine (1-NM-PP1) on the right. **g** In vitro kinase assay with wild-type and the analog-sensitive CDKA;1 (CDKA;1^F80G^) kinases using CYCD3;1 as a cyclin partner and histone H1 as a generic substrate. Inhibition of wild-type (*left*) and the analog-sensitive CDKA;1 (*right*) kinases with 0, 1, and 10 μM of the PP1 derivative 1-NM-PP1. Proteins were subjected to SDS-PAGE after the kinase reaction with [γ-^32^P]-ATP as a phosphate donor and stained with Coomassie brilliant blue R-250 demonstrating equal loading of the substrate. Mock was treated with 0.1 % (v/v) DMSO, the solvent of 1-NM-PP1. *Abbreviations*: p-H1 for radio-labeled histone H1 resulting from kinase assays with radio-labeled ATP, H1 for histone H1. Chemical structures in this figure were drawn with MarvinSketch, version 5.0.02 (ChemAxon, Hungary)

One of the most successful procedures to detect kinase targets in yeast and animals has been a chemical genetics approach relying on the observation that a large hydrophobic or polar residue in the ATP-binding pocket of the kinase domain can – at least in some cases – be mutated to a smaller amino acid, such as glycine (G), without largely altering kinase kinetics [18] (Fig. 1a). The exchange of this ‘gatekeeper’ amino acid increases the size of the ATP-binding pocket so that enlarged (‘bulky’) ATP analogues such as N^6^-benzyladenosine-5′-O-triphosphate (6-Bn-ATP) and N^6^-(2-phenylethyl)adenosine-5′-O-triphosphate (N6-PhEt-ATP) can be used in phospho-transfer reactions. Moreover, bulky kinase inhibitors that are derived from 4-amino-1-*tert*-butyl-3-phenylpyrazolo[3,4-*d*]pyrimidine (PP1), e.g. 4-amino-1-*tert*-butyl-3-(1′-naphthylmethyl)pyrazolo[3,4-*d*]pyrimidine (1-NM-PP1) can be used to specifically inhibit these analog-sensitive kinases [19, 20].

The use of analog-sensitive kinases has been pioneered in particular by the laboratory of Kevan Shokat and such engineered kinases have become a very powerful tool to study many biological problems, for instance in cell-cycle regulation, by either identifying kinase substrates or by modulating their function during the cell cycle [6, 21–23]. Notably, the tunability of analog-sensitive kinases allows the replacement of temperature-sensitive mutants, which have been widely used in the past but often produced many artifacts due to the high (not physiological) temperature needed for their inactivation, for instance when studying meiosis [24, 25].

Analog-sensitive kinases have also been successfully used in plants to study different signaling processes including MAP-kinases, calcium-dependent protein kinases, and the protein kinase Pto that confers resistance of tomato plants (*Solanum lycopersicum*) against the bacterium *Pseudomonas syringae* [26–30].

Here, we adopted this chemical genetics strategy to study the plant cell cycle and generated an analog-sensitive version of CDKA;1 that largely complemented a *cdka;1* mutant. Application of a PP1 analog as a kinase inhibitor was found to specifically reduce the growth of these analog-sensitive *cdka;1* mutant plants. Using then a two-dimensional differential gel electrophoresis (2D-DIGE) approach involving bulky ATP derivatives, we performed here a pilot screen and identified a list of putative CDKA;1 substrates of which five selected substrates were confirmed by kinase assays. These substrates indicate novel routes how growth and cell proliferation could be linked to metabolism and physiology during plant development.

## Results

### Generation of an analog-sensitive variant of CDKA;1

*Arabidopsis* CDKA;1 shares a high degree of sequence similarity with human Cdk1, Cdk2, and Cdk3 as well as the yeast Cdc2 and CDC28 kinases (Additional file 1: Figure S1A). When we modeled CDKA;1 onto a known crystal structure of human Cdk2, all of the important structural elements of Cdk2 could be matched in CDKA;1, e.g. the T-loop (involved in substrate binding) and the P-loop (functioning in activity regulation), in accordance with previous reports showing that the molecular mechanistics of CDKA;1 function are conserved (Additional file 1: Figure S1B) [31–33].

This model indicated that the conserved amino acid Phenylalanine (Phe/F) 80 could function as a gatekeeper residue in restricting the size of the putative ATP binding pocket of CDKA;1, consistent with a prediction deposited in the kinase sequence database (http://sequoia.ucsf.edu/ksd/) [34] (Table 1; Fig. 1b; Additional file 1: Figure S1A). Therefore, we substituted Phe80 to Glycine (Gly/G) (CDKA;1^F80G^) with the aim to increase the size of the pocket allowing the use of bulky ATP derivatives, such as N^6^-(2-phenylethyl)adenosine-5′-O-triphosphate (N6-PhEt-ATP), in phosphorylation reactions (Fig. 1c, d). To evaluate the biochemical activity of the CDKA;1^F80G^ protein, we performed in vitro kinase assays with bacterially expressed proteins using a bulky ATP, i.e. N6-PhEt-ATP, and histone H1 that is typically used as a generic substrate to measure Cdk activity [35, 36]. Although the CDKA;1^F80G^ kinase activity was decreased compared to the wild-type kinase, only CDKA;1^F80G^ could catalyze the bulky ATP demonstrating a high level of specificity that is needed for further substrate identification procedures (Fig. 1e).

**Table 1:** Structure-based sequence alignment of CDKs for the chemical-genetic approach

Kinase	ß-sheets														
	ß2			ß3					ß4			ß5			
Cdk1 (H.s.)	V	**V**	Y	V	**A**	M	**K**	K	I	**V**	S	Y	**L**	**I**	**F**_**80**_
Cdk2 (H.s.)	V	**V**	Y	V	**A**	L	**K**	K	I	**V**	K	Y	**L**	**V**	**F**_**80**_
Cdk3 (H.s.)	V	**V**	Y	V	**A**	L	**K**	K	I	**V**	R	Y	**L**	**V**	**F**_**80**_
Cdc2 (S.p.)	V	**V**	Y	V	**A**	M	**K**	K	C	**V**	R	Y	**L**	**V**	**F**_**84**_
CDC28 (S.c.)	V	**V**	Y	V	**A**	L	**K**	K	I	**V**	R	Y	**L**	**V**	**F**_**88**_
CDKA;1 (A.t.)	V	**V**	Y	I	**A**	L	**K**	K	I	**V**	K	Y	**L**	**V**	**F**_**80**_

*Homo sapiens* (H.s.), *Schizosaccharomyces pombe* (S.p.), *Saccharomyces cerevisiae* (S.c.), *Arabisopsis thaliana* (A.t.). Bold letters mark residues contacting ATP in the active site. Numbers indicate the positions of the respective residues in the protein. The “gatekeeper” positions are numbered

An enlarged ATP-binding pocket usually confers sensitivity towards bulky derivatives of general kinase inhibitors such as PP1 (Fig. 1f). We therefore asked if CDKA;1^F80G^ showed analog-sensitivity in vitro against the bulky PP1 derivate 1-NM-PP1 (Fig. 1f). Treatment of 1-NM-PP1 inhibited the kinase activity of CDKA;1^F80G^, but not of the wild-type CDKA;1, in a dose-dependent manner (Fig. 1g).

**Fig. 2:**
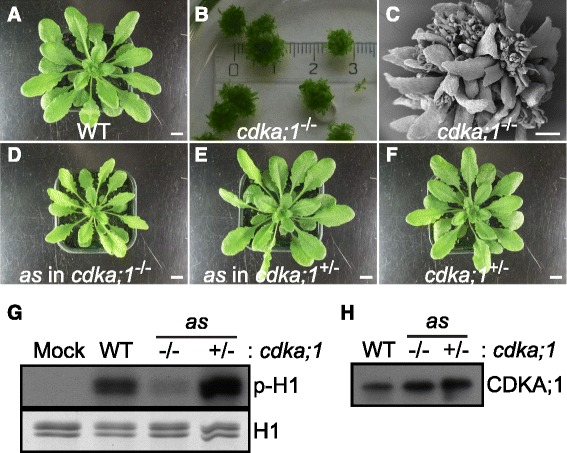
Expression of *cdka;1-as* largely restores the defects of *cdka;1* mutants. **a** Wild type rosette plants, approximately 1 month after sowing. Scale bar: 1 cm. **b** The *cdka;1* homozygous mutants are extremely dwarf and can only grow on agar or in liquid media due to the absence of a functional root. Ruler scale is at cm. **c** Scanning electron micrograph of a 3 month-old *cdka;1* homozygous mutant plant seen in **b**. Scale bar: 1 mm. **d** The expression of the *cdka;1-as* (*CDKA;1* ^*F80G*^) mutant largely rescues the development of homozygous *cdka;1* null mutants that develop a root and can grow on soil. Plant shown was planted the same time as the wild-type control in panel **a**. Scale bar: 1 cm. **e** The expression of *cdka;1-as* does not confer a gain-of-function effect as seen in plants that contain the as allele in the heterozygous *cdka;1* mutant background. Plant shown was planted the same time as the wild-type control in panel **a**. Scale bar: 1 cm. **f** Heterozygous *cdka;1* mutants as a control. Plant shown was planted the same time as the wild-type control in panel **a**. Scale bar: 1 cm. **g** p13^Suc1^-associated protein kinase activity purified from wild-type plants (WT), *cdka;1-as* plants (in homozygous *cdka;1* ^*−/−*^ (−/−), and heterozygous *cdka;1* ^*+/−*^ (+/−) mutant background) or buffer (Mock), respectively, against bovine histone H1 as a generic substrate. Proteins were subjected to SDS-PAGE after the kinase reaction and stained with Coomassie brilliant blue R-250 demonstrating equal loading of the substrate. *Abbreviations*: p-H1 for radio-labeled histone H1 resulting from kinase assays with radio-labeled ATP, H1 for histone H1. **h** Protein blot analysis extracts from the wildtype plant (*left*), *cdka;1-as* plant in *cdka;1* ^*−/−*^ (*middle*) and *cdka;1* ^*+/−*^ (*right*) background, respectively, were probed with the antibody raised against the PSTAIRE cyclin-binding motif demonstrating comparable level of CDKA;1 in the indicated genotypes

A major aim of this study was to generate an in vivo tool to identify kinase substrates and modulate kinase activity in the developmental context of a multicellular organism. To test the biological activity of CDKA;1^F80G^, a cDNA encoding the mutant version was placed under the control of the endogenous *CDKA;1* promoter that has been previously used in a transgenic approach to express the wild-type CDKA;1 cDNA resulting in a complete rescue of *cdka;1* null mutant plants [37]. Since null mutants of *CDKA;1* are sterile and extremely dwarfed [14] (Fig. 2a, b, c), heterozygous *cdka;1* mutants were transformed with the *PRO*_*CDKA;1*_*:CDKA;1*^*F80G*^ construct*.* Importantly, we obtained wild-type looking plants that were homozygous *cdka;1* mutant in the progeny of the transformed heterozygous *cdka;1* mutant plants (Fig. 2d). These plants were found to contain the *PRO*_*CDKA;1*_*:CDKA;1*^*F80G*^ construct (hereafter referred to as *cdka;1-as* plants) confirming the biological activity of the *CDKA;1*^*F80G*^ variant. Closer inspection showed that rescue was not 100 % since *cdka;1-as* plants were slightly smaller than wild-type plants as they grew older (Fig. 2a, d). However, *cdka;1-as* mutant plants grew larger than previously identified weak loss-of-function *cdka;1* mutants [31, 32] (data not shown). The *cdka;1-as* construct did also not confer a dominant negative effect since heterozygous *cdka;1* mutants containing the construct grew similar to the untransformed plants consistent with the conclusion that *CDKA;1*^*F80G*^ is functional *CDKA;1* allele, albeit with reduced activity (Fig. 2e, f).

Next, we assessed kinase activity of *cdka;1-as* by extracting CDK-cyclin complexes from extracts of inflorescences of each genotype with beads coated with p13^Suc1^ that is known to bind to *Arabidopsis* CDKs including CDKA;1 [38]. Consistent with the reduced plant growth of *cdka;1-as* and reduced kinase activity levels of *CDKA;1*^*F80G*^ in vitro, we found that p13^Suc1^-associated kinase activity (with regular, i.e. non-bulky ATP) from these plants was decreased in comparison to that of wild-type plants using bovine histone H1 as a generic substrate (Fig. 2g, h).

Taken together, the F80G gatekeeper mutation of CDKA;1 diminishes kinase activity in vitro and in vivo. A reduction in kinase activity has been reported for other gatekeeper mutant CDK versions and hence the here-generated version was in the expectation range of an analog-sensitive kinase [39]. Importantly, the *Arabidopsis* analog-sensitive CDKA;1 version CDKA;1^F80G^ has sufficient activity to support growth and development largely resembling the wildtype, this was not the case with hypomorphic alleles described previously [31, 32] indicating an overall stronger activity in vivo. To our knowledge the here-generated *cdka;1-as* line is the first analog-sensitive CDK that can be studied in the developmental context of a multicellular organisms and hence represents a novel tool to modulate CDKA;1 activity and potentially identify novel CDK substrates.

### Modulating plant growth

**Fig. 3:**
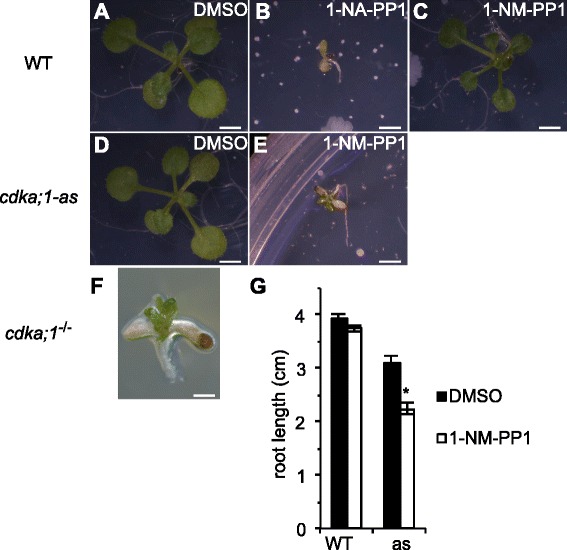
Modulation of plant growth in vivo. **a** Wild-type control plants grown for 2 weeks on MS plates containing the solvent DMSO and no bulky kinase inhibitor. Scale bar: 4 mm. **b** Wild-type plants grown for 2 weeks on MS plates supplemented with 100 μM 1-NA-PP1 die. Scale bar: 4 mm. **c** Wild-type plants grown for 2 weeks on MS plates supplemented with 100 μM 1-NM-PP1 are smaller than wild-type plants grown without the inhibitor but survive. Scale bar: 4 mm. **d** *cdka;1* ^*−/−*^ *PRO*_*CDKA;1*_ *:CDKA;1* ^*F80G*^ (*cdka;1-as*) grown for 2 weeks on MS plates containing the solvent DMSO and no bulky kinase inhibitor are slight reduced in their size in comparison with the wild-type control plants, see also Fig. 2a, d. Scale bar: 4 mm. **e** *cdka;1-as* grown for 2 weeks on MS plates supplemented with 100 μM 1-NM-PP1 is severely compromised with arrested root development. Scale bar: 4 mm. **f** A homozygous *cdka;1* ^*−/−*^ seedling grown on a MS plate for 2 weeks after germination shows the typical phenotype of loss of CDKA;1 function with halted root development and only a few and tiny leaves being formed. Scale bar: 1 mm. **g** Root length measurement of the seedlings of wild type (Col-0) and *cdka;1-as* (*as*) grown on MS plates supplemented with 10 μM 1-NM-PP1 for 7 days after germination. Error bars represent the SE. A statistical significant change between the mock and 1-NM-PP1 treatment is marked by an asterisk above the bar (t-test, *P* = 0.0002 <0.05)

As a first test of the usability of the analog-sensitive mutant versions, we aimed to phenocopy the *cdka;1* null mutant phenotype when applying bulky kinase inhibitors. To this end we used two different inhibitors, 1-NA-PP1 or 1-NM-PP1 (Fig. 1g), that have been successfully used in yeast and mammalian systems. We started with the application of high concentrations, i.e. 100 μM, to completely abolish CDKA;1 activity and generate chemically induced loss-of-function mutants [7, 40]. Although the treatment of *Arabidopsis* seedlings with 100 μM 1-NA-PP1 was reported previously [27], the application of this compound severely affected the development of wild-type plants under our growth conditions and was therefore not further considered as a chemical CDK inhibitor for *cdka;1-as* (Fig. 3a, b). Wild-type plants grown on agar plates containing 100 μM 1-NM-PP1 survived although they were slightly reduced in their growth at this high concentration (Fig. 3a, c). In contrast, the treatment of *cdka;1-as* plants with 100 μM 1-NM-PP1 severely reduced their growth resembling homozygous *cdka;1* mutants (Fig. 3d, e, f).

Next, we asked whether plant growth could be modulated by applying a lower concentration of 1-NM-PP1. To assay this, we monitored root growth based on the observation that *Arabidopsis* root growth is in particular sensitive to CDKA;1 levels [14, 32, 41]. The growth of mock-treated wild-type plants was not significantly different from wild-type plants grown on agar plates containing 10 μM 1-NM-PP1 (t-test, *P* = 0.2 >0.05, Fig. 3g). While the roots of mock-treated *cdka;1-as* plants had approximately 80 % of the length of mock-treated wild-type plants, treatment with 10 μM 1-NM-PP1 significantly reduced their size by nearly additional 25 % in contrast to the root growth arrest observed at 100 μM 1-NM-PP1 (t-test, *P* = 0.0002 <0.05; Fig. 3e, g). Thus, growth of the *cdka;1-as* plants generated here can be chemically modulated in vivo setting a base for a detailed analysis and assessment of cell-cycle activity during organ growth and development in the future.

### Identification of putative CDK substrates by 2D-DIGE

**Fig. 4:**
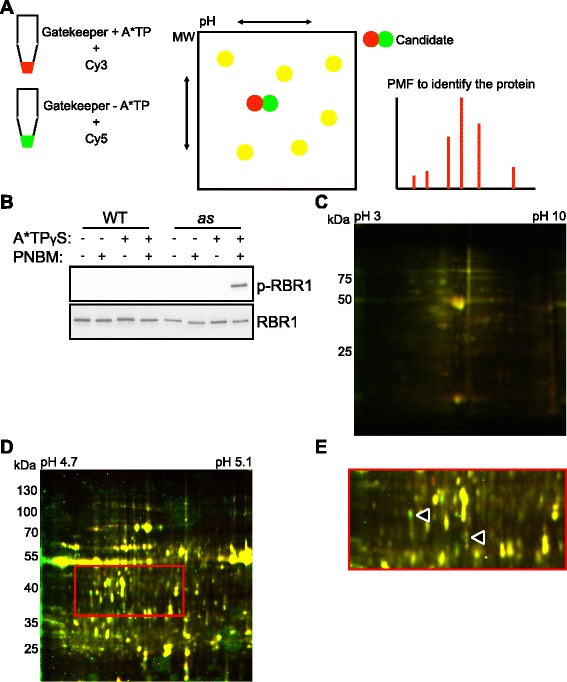
Identification of CDKA;1 substrates by 2D-DIGE. **a** Strategy for identifying the kinase substrate by 2D-DIGE as applied here. For details see descriptions in the text. **b** In vitro kinase assay using wild-type and the analog-sensitive CDKA;1 (CDKA;1^F80G^) kinases together with CYCD2;1 as a cyclin partner using GST-RBR1-His_6_ as a substrate. After the kinase reaction with N6-PhEt-ATP-γ-S as a phospho-donor, proteins were alkylated with PNBM and were subjected to SDS-PAGE and transferred to a membrane. Thiophosphorylated RBR1 was detected with anti-thiophosphate ester antibody (*top*) and protein blot with anti-GST antibody (*bottom*) is showing an equal loading of the substrate. Mock was treated with 5 %(v/v) DMSO, the solvent of PNBM. Abbreviations: PNBM, p-nitrobenzyl mesylate, p-RBR1 for thiophosphorylated RBR1 resulting from kinase assays with N6-PhEt-ATP-γ-S. **c** A representative 2D-DIGE analysis. Protein extracts from wild-type seedlings incubated in the presence or absence of N6-PhEt-ATP-γ-S were labeled separately with Cy3 (532 nm, *red*) and Cy5 (635 nm, *green*), and proteins were then separated in the same gel in two dimensions and visualized by laser scanning. Most of the proteins from each treatment were focused similarly indicating a very low background level using N6-PhEt-ATP-γ-S. **d** A representative 2D-DIGE analysis. Protein extracts from *cdka;1-as* inflorescences incubated in the presence or absence of N6-PhEt-ATP-γ-S were labeled separately with Cy3 (532 nm, *red*) and Cy5 (635 nm, *green*). Proteins were separated and analyzed as in **c. e** Magnified image of C, showing that some spots were focused differently (*arrow heads*)

A second goal of constructing *cdka;1-as* plants was to identify novel CDKA;1 substrates since so far only a handful of CDKA;1 targets are known in plants versus over 300 substrates of CDC28/Cdk1 that have been identified in yeast [7, 10, 42]. To this end we followed a strategy based on 2D-DIGE to identify putative CDK phospho-targets. The basis for this approach is the fact that post-translational modifications such as phosphorylation usually affect the isoelectric point and molecular weight of the proteins, by which their electrophoretic mobility is altered in the gel (Fig. 4a).

First, we asked if CDKA;1^F80G^ can catalyze the bulky ATP derivative N6-PhEt-ATP-γ-S. The rationale of using a thio-ATP variant was to limit the reversal of the kinase reaction since thio-phosphorylated proteins have been shown to be less efficiently dephosphorylated by phosphatases [43–45]. To detect thio-phosphorylated substrates, we followed a previously presented strategy that is based on the alkylation of thio-phosphorylated serine and threonine (or tyrosine) residues creating thereby an epitope for a thiophosphate ester-specific antibody [46]. For this experiment, we used the *Arabidopsis* Retinoblastoma homolog RETINOBLASTOMA RELATED 1 (RBR1) as a native substrate since previous studies have indicated that it is one if not the most important CDKA;1 substrate in vivo [14]. For this purpose, recombinant full-length RBR1 protein fused to a dual tag to facilitate purification (GST-RBR1-His_6_) was generated in *E. coli*. The purified recombinant protein was incubated with wild-type CDKA;1 or CDKA;1^F80G^ and N6-PhEt-ATP-γ-S followed by direct alkylation with p-nitrobenzylmesylate (PNBM). Protein blots were performed to detect thiophosphorylated RBR1 with the anti-thiophosphate ester antibody, raised against a p-nitrobenzylthiophosphate ester. The signal was detected only in assays with CDKA;1^F80G^ (Fig. 4b), indicating that CDKA;1^F80G^ can use N6-PhEt-ATP-γ-S as a thiophosphate donor.

To determine a possible background (false positive) label when using N6-PhEt-ATP-γ-S, we incubated extracts from wild-type plants exchanging the buffer to remove the endogenous ATP (see the detail in materials and methods) and in the presence or absence of N6-PhEt-ATP-γ-S and labeled both fractions with Cy3 (532 nm, red) and Cy5 (635 nm, green), respectively. The two extracts were then separated in the same gel using 2D-DIGE and visualized by laser scanning. This experiment showed that the great majority of proteins was similarly focused indicated by the lack of separation of red and green dots. Hence, we concluded that there is only a very low level of unspecific use of N6-PhEt-ATP-γ-S by endogenous *Arabidopsis* kinases paving the road for the use of analog-sensitive kinases to identify substrates (Fig. 4c).

In the next step, we followed the same experimental procedure using extracts from *cdka;1-as* mutants (Fig. 4a, d, e). Putative substrates can be identified by differentially colored spots and non-overlapping but closely positioned spots on the gel. Detection relies on our observation that CDK targets in the protein extracts supplemented with the bulky ATP can almost exclusively only be phosphorylated by CDKA;1^F80G^ (as shown by the high specificity of CDKA;1-AS and the low background level in wildtype samples using N6-PhEt-ATP-γ-S) resulting in an altered electrophoretic mobility. In contrast, proteins that have the same migration behavior in extracts with and without the bulky ATP derivative will appear as yellow spots resulting from the overlay of red and green colors (Fig. 4d, e).

By peptide-mass fingerprinting, we could then identify a total of 20 candidates that showed different migration patterns representing putative CDKA;1 substrates (Table 2). These potential substrates mapped into many different developmental and physiological pathways potentially linking CDK activity with many core cellular functions.

**Table 2:** Candidates of CDKA;1 substrates identified in this study

Gene Locus	Identification	[R/K]xL^a^	[S/T]P^b^	[S/T]Px[R/K]^c^	CYCD2^d^	PhosPhAt^e^
At1g13440	GLYCERALDEHYDE-3-PHOSPHATE DEHYDROGENASE C2 (GAPC2)	2	–	–	n.d.	–
At1g35720	ANNEXIN 1 (ANNAT1)	4	–	–	n.d.	–
At1g52400	BETA GLUCOSIDASE 18 (BGLU18)	5	5	–	n.d.	–
At1g53240	MITOCHONDRIAL MALATE DEHYDROGENASE 1 (mMDH1)	1	2	–	+	–
At1g54100	ALDEHYDE DEHYDROGENASE 7B4 (ALDH7B4)	4	2	–	+	–
At2g14260	PROLINE IMINOPEPTIDASE (PIP)	3	1	–	+	–
At2g31390	pfkB-like carbohydrate kinase family protein	3	–	–	+	–
At2g36530	LOW EXPRESSION OF OSMOTICALLY RESPONSIVE GENES 2 (LOS2)	1	–	–	n.d.	–
At2g39730	RUBISCO ACTIVASE (RCA)	3	–	–	n.d.	–
At3g09440	Heat shock protein 70 (Hsp 70) family protein	1	2	–	n.d.	–
At3g09820	ADENOSINE KINASE 1 (ADK1)	3	2	–	n.d.	–
At3g51260	20S PROTEASOME ALPHA SUBUNIT PAD1 (PAD1)	2	2	–	n.d.	–
At3g52390	TatD related DNase	2	1	–	n.d.	–
At4g25240	SKU5 SIMILAR 1 (SKS1)	3	4	1	n.d.	–
At4g38220	AQUAPORIN INTERACTOR (AQI)	5	6	–	n.d.	–
At4g38970	FRUCTOSE-BISPHOSPHATE ALDOLASE 2 (FBA2)	2	4	1	–	2
At5g14200	ISOPROPYLMALATE DEHYDROGENASE 1 (IMD1)	2	2	1	+	1
At5g17920	METHIONINE SYNTHESIS 1 (ATMS1)	8	2	–	n.d.	1
At5g19440	NAD(P)-binding Rossmann-fold superfamily protein	4	2	–	n.d.	–
At5g26000	THIOGLUCOSIDE GLUCOHYDROLASE 1 (TGG1)	3	3	–	n.d.	–
At5g51830	pfkB-like carbohydrate kinase family protein	3	–	–	n.d.	–
At5g62790	1-DEOXY-D-XYLULOSE 5-PHOSPHATE REDUCTOISOMERASE (DXR)	2	2	–	n.d.	–

^a^Putative cyclin binding motif; [RK].L. [0,1][FYLIVMP]

^b^Putative minimal motif in CDK substrates at site of phosphorylation

^c^Putative maximal motif in CDK substrates at site of phosphorylation

^d^Phosphorylatin was confirmed by CDKA;1-CYCD2;1 complexes. +; positive -; negative *n.d.* not determined

^e^Peptides phosphorylated at [S/T]P sites were found in the PhosPhAt 4.0 data base

### Confirmation by in vitro kinase assays

To test whether the proteins identified by a differential migration pattern in 2D-DIGE are indeed substrates of CDKA;1, we performed in vitro kinase assays. We first generated His:GST-tagged versions of the following six randomly chosen proteins of the list of 20 potential substrates and expressed them in *E. coli*: ALDH7B4 (At1g54100), FBA2 (At4g38970), IMD1 (At5g14200), mMDH1 (At1g53240), pfkB-like (At2g31390), and PIP (At2g14260) (Table 2).

**Fig. 5:**
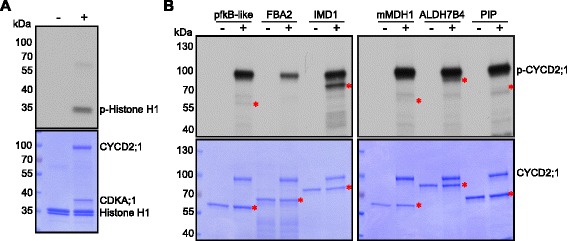
In vitro kinase assay against candidate proteins. **a** Histone H1 kinase assay. Proteins were subjected to SDS-PAGE after the kinase reaction with (+) or without (−) CDKA;1-CYCD2;1 complexes and stained with Coomassie brilliant blue R-250 demonstrating equal loading of the substrate. Abbreviations: p-histone H1 for radio-labeled histone H1 resulting from kinase assays with radio-labeled ATP. **b** In vitro kinase assays against candidate proteins. Proteins were subjected to SDS-PAGE after the kinase reaction with (+) or without (−) CDKA;1-CYCD2;1 complexes and stained with Coomassie brilliant blue R-250. Asterisks in the autoradiograph (*top*) show phosphorylated substrates, in the Coomassie stain (*bottom*) demonstrate equal loading of the recombinant substrate candidates, respectively. Abbreviations: p-CYCD2;1 for radio-labeled CYCD2;1 resulting from autophosphorylation by kinase assays with radio-labeled ATP

The purified proteins were then used in in vitro kinase assays with CDKA;1-CYCD2;1, a complex that has previously been shown to build a functional dimer (Fig. 5a) [47]. Out of the six proteins tested, all but FBA2 were phosphorylated in our in vitro assay (Fig. 5b). We can currently not exclude that FBA2 is also a CDK substrate since the cyclin unit is known to play a key role in substrate specificity and we tested here only one out of more than 30 theoretically possible CDKA;1-cyclin combinations in *Arabidopsis*. Moreover, FBA2 has been shown in other large-scale experiments to be phosphorylated at one short and one long CDK consensus site (Table 2; PhosPhAt 4.0, http://phosphat.uni-hohenheim.de/) [48, 49]. Importantly, the observation that five out of six proteins could be phosphorylated by CDKA;1-CYCD2;1 in vitro provides biochemical evidence that the 2D-DIGE strategy in combination with analog-sensitive kinase variants allows the identification of CDK substrates.

**Fig. 6:**
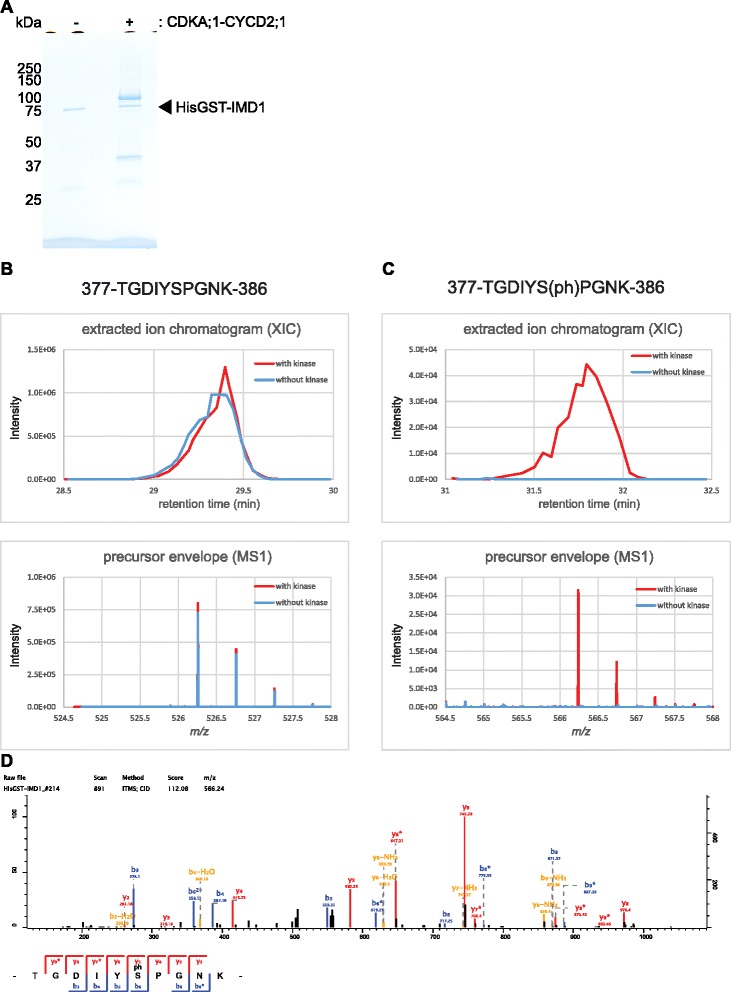
Identification of phosphorylation sites of IMD1 treated with CDKA;1-CYCD2;1 complexes in vitro. **a** Gel image of HisGST-IMD1 subjected to mass spectrometry analyses. The IMD1 proteins were treated without (−) and with (+) CDKA;1-CYCD2;1. The proteins were separated by SDS-PAGE after the kinase reaction and stained with coomassie brilliant blue. **b** Mass chromatograms (*top*) of the selected peptide and mass spectrum (*bottom*) of the peptide. Corresponding to (**c**), the non-phosphorylated peptide 377-TGDIYSPGNK-386 was detected in the both samples of IMD1 treated with (*red*) and without (*blue*) CDKA;1-CYCD2;1 complexes. **c** Mass chromatograms (*top*) of the selected peptide and mass spectrum (*bottom*) of the peptide. The phosphopeptide 377-TGDIYS(ph)PGNK-386 was detected only in the sample of IMD1 treated with CDKA;1-CYCD2;1 (*red*), but not in the sample without kinase (*blue*). S(ph), in the peptide sequence, indicates the phosphorylated serine. **d** MS/MS spectra of a phosphopeptide (377-TGDIYS(ph)PGNK-386) from IMD1 treated with CDKA;1-CYCD2;1. The b and y ion series represent fragment ions containing the N- and C-termini of the peptide, respectively. Mass chromatogram (**b** and **c**, *top*) is given by plotting the x-axis as the retention time and the y-axis as the ion peak intensity. Mass spectrum (**b** and **c**, *bottom*) is given by plotting the x-axis as the mass-to-charge ratio (m/z) and the y-axis as the ion peak intensity

**Fig. 7:**
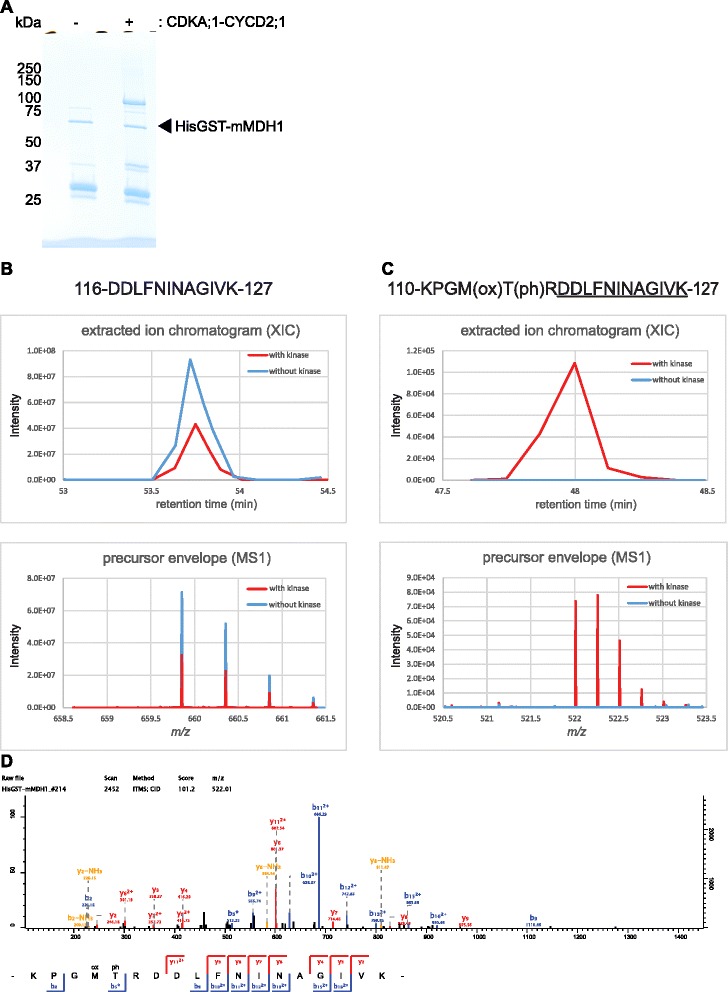
Identification of phosphorylation sites of mMDH1 treated with CDKA;1-CYCD2;1 complexes in vitro. **a** Gel image of HisGST-mMDH1 subjected to mass spectrometry analyses. The mMDH1 proteins were treated without (−) and with (+) CDKA;1-CYCD2;1. The proteins were separated by SDS-PAGE after the kinase reaction and stained with coomassie brilliant blue. **b** Mass chromatograms (*top*) of the selected peptide and mass spectrum (*bottom*) of the peptide. Corresponding to (**c**), the non-phosphorylated peptide 116-DDLFNINAGIVK-127 was detected in the both samples of mMDH1 treated with (*red*) and without (*blue*) CDKA;1-CYCD2;1 complexes. **c** Mass chromatograms (*top*) of the selected peptide and mass spectrum (*bottom*) of the peptide. The phosphopeptide 110-KPGM(ox)T(ph)RDDLFNINAGIVK-127 was detected only in the sample of mMDH1 treated with CDKA;1-CYCD2;1 (*red*), but not in the sample without kinase (*blue*). T(ph) and M(ox), in the peptide sequence, indicate the phosphorylated threonine and the oxidized methionine, respectively. The underline in the peptide sequence indicates the peptide sequence found in (**b**). **d** MS/MS spectra of a phosphopeptide (110- KPGM(ox)T(ph)RDDLFNINAGIVK-127) from mMDH1 treated with CDKA;1-CYCD2;1. The b and y ion series represent fragment ions containing the N- and C-termini of the peptide, respectively. Mass chromatogram (**b** and **c**, *top*) is given by plotting the x-axis as the retention time and the y-axis as the ion peak intensity. Mass spectrum (**b** and **c**, *bottom*) is given by plotting the x-axis as the mass-to-charge ratio (m/z) and the y-axis as the ion peak intensity

To further characterize the CDKA;1 phosphorylation sites, we subjected as an example the two here-identified CDKA;1 substrates IMD1 and mMDH1 to phospho-mass- spectrometry analyses. To this end, sample for both proteins were either treated CDKA;1-CYCD2;1 or not prior to their mass analyses (Figs. 6a and 7a). The phosphopeptide 377-TGDIYS(ph)PGNK-386 (with S(ph) indicating the phosphorylated serine 382 matching the Cdk consensus sequence) was detected only in the sample of IMD1 treated with CDKA;1-CYCD2;1 while the non-phosphorylated peptide 377-TGDIYSPGNK-386 was detected in the both samples of IMD1 (Fig. 6b,c,d).

Similarly, the phosphorylated peptide 110-KPGM(ox)T(ph)RDDLFNINAGIVK-127 (with T(ph) indicating the phosphorylated threonine 114 in a non-consensus Cdk site) was only found in the sample of mMDH1 treated with CDKA;1-CYCD2;1 activity (Fig. 7b,c,d). However, there was no corresponding match to the non-phosphorylated peptide 110-KPGM(ox)TRDDLFNINAGIVK-127 in both samples. We speculated that if the Thr in front of the Arg in this peptide is phosphorylated, trypsin can hardly cut the peptide after the Arg. As an alternative, we henced measured the peptide 116-DDLFNINAGIVK-127 in both samples demonstrating the specificity of the phosphorylated peptide in sample treated with CDK activity.

## Discussion

The identification of kinase substrates remains one of the major challenges for many biological questions. One of the main reasons for our lack of knowledge of substrates is the intrinsically transient nature of the enzyme–substrate interaction, i.e. the “kiss and run” mechanism. Another reason is the high degree of structural and mechanistic similarities of protein kinases that all belong into one large superfamily [4]. Here we have adopted a chemical genetics procedure, which has been very successfully applied in yeast as well as in animals [18, 20], and generated an analog-sensitive variant of the major cell-cycle kinase CDKA;1 in the flowering plant *Arabidopsis thaliana*. To our knowledge, this is the first example in which an analog sensitive cell-cycle kinase has been generated and studied at an organismic level of a multicellular organism. This version has allowed us to modulate plant growth in vivo and identify novel CDKA;1 substrates, thus representing a useful tool to analyze plant cell-cycle control.

### Substrate identification by 2D-DIGE

Several methods have been employed over the last years to reveal kinase substrates. Starting from the identification of kinase targets in specialized interaction assay, such a yeast-two-hybrid or bimolecular complementation assays [50, 51]. A breakthrough was the development of chemical genetics approaches together with proteome-wide identification of phosphorylated proteins that tremendously promoted our understanding of phosphorylation events [17, 18, 20]. These approaches, which have been pioneered in yeast in human cell culture systems, are also now applied in plants allowing eventually a cross-kingdom proteome comparison of phosphorylation levels and states [52].

The chemical genetics approach to identify kinase substrates typically makes use of an antibody to detect thiophosphorylated proteins. In contrast, we have combined a 2D-DIGE approach with analog-sensitive kinases. While the antibody-based technique is likely more specific, the 2D-DIGE approach is also more cost-effective. Another potential benefit of the 2D-DIGE method is that, at least in theory, the resolution can be modulated by using different gels (for the 1st and 2nd dimension). However, we often did not find both distinct green and red but rather only a red or a green spot in our analyses. Hence, there is room for improvement of this method. At the same time, the identification of Cdk substrates in plants is also still at the beginning and will require much more work and complementary experiments in the future. None-the-less, we could identify by our 2D-DIGE approach several potential Cdk substrates, which we could subsequently confirm by in vitro kinase assays.

### CDK substrates within and outside of the cell cycle

CDKs are proline-directed serine/threonine protein kinases and their substrates often contain the phosphorylation signature [Ser/Thr]-Pro-X-[Lys/Arg], in which the phosphorylated amino acid (S or T) is typically followed by a P and a positively charged amino acid at the position +3 and/or +4 that interacts with the negatively charged phosphate group in the T-loop of the kinase (X; any amino acid) [53]. Consistently, the majority of the here-identified CDK targets and substrate candidates contained a consensus site, i.e. three proteins with the long and 13 with at least one short consensus site (Table 2). In addition, cyclins mediate substrate interaction since CDK substrates often contain a short [R/K]xL (also called Cy) motif that interacts with a small hydrophobic patch on the surface of the cyclin [54]. However, several *bona fide* substrates in yeast have been found to harbor no [S/T]P motif [6, 51]. An example from this study is the putative substrate At5g51830 that encodes a ribokinase protein (Table 2) and the mapped phospho-site in the CDKA;1 substrate mMDH1 (Fig. 7). Thus, the presence or absence of a CDK consensus phosphorylation site is not an unambiguous indication that the respective protein is indeed a CDK substrate making forward experimental assays such as the here-applied strategy necessary.

The best-known CDK substrates are components of the cell-cycle machinery such as the pre-replication complex. In the here-presented pilot study, extracts from inflorescences were used in which proliferating cells represent only a minority of cell types. Moreover, even in proliferating cells cell-cycle regulators are typically not highly abundant proteins. Thus, it is not surprising that we did not identify cell-cycle regulators as targets. For future approaches, 2D-DIGE experiments can be performed with selected tissues at specific developmental time points to enrich for specific classes of CDK substrates. In addition, the use of cell cultures that can be chemically synchronized represents a possibility to enrich for CDKA;1 substrates in different phases of the cell cycle. Notably, proteins with a clear function outside of the cell cycle can also be found among the more than 300 CDC28 substrates in the *S. cerevisiae* proteome [6–8]. Correspondingly, more than 1000 genes have been found to be expressed in a cell-cycle phase dependent manner in *Arabidopsis* cell culture also indicating a central function of the cell cycle in orchestrating many cellular functions in plants outside of DNA replication and mitosis [55].

Interestingly, the activity of the here-identified mMDH1 that encodes a mitochrondrial malate dehydrogenase was identified to oscillate in synchronized cultures of *Euglena* (single-celled flagellate protists) and correlated with cell-cycle activity and the light regime [56]. Moreover, malate dehydrogenases have been associated with cell-proliferation control since MDH1 was found to serve as a transcriptional co-activator of p53 in mammals by moving to the nucleus and binding to the promoter of p53-downstream genes. Thus, MDH1 contributes to the p53-mediated cell-cycle arrest and cell death in response to glucose deprivation [57]. However, the possible role of mMDH1 phosphorylation in *Arabidopsis* is not clear yet. mMDH1 contains two minimal CDK phosphorylation sites of which one (Thr66) is a predicted phosphorylation site in the PhosPhAt database. However, the here identified non-consensus CDKA;1 phosphorylation site at Thr114 has not been deposited in the PhosPhAt database (http://phosphat.uni-hohenheim.de) [48, 49].

The nutritional status of a cell, especially regarding carbohydrates, is well-known to be an important regulator of the cell cycle [58]. Thus, feedback mechanisms from the cell cycle to the metabolic state are likely to have evolved. Another potential link between sugar metabolism and the cell cycle is represented from our work by phosphorylation of the ribokinase At2g31390 (EC 2.7.1.4) by CDKA;1 that belongs to the pfkB-like carbokinase family, a large yet poorly characterized group within the ribokinase family [59]. Furthermore, a second pfkB-like carbokinase family protein (At5g51830) is among the putative but not yet biochemically confirmed substrates. Carbohydrate kinase-like proteins have been reported to serve both regulatory as well as direct catalytic functions. Members include two *Arabidopsis* ADENOSINE KINASES (ADK) and reduced ADK levels result in growth defects [60], reduced root gravitropism [61], and defects linked to altered cytokinin levels [62].

The here-identified CDKA;1 target proline iminopeptidase (PIP; E.C. 3.4.11.5) catalyzes the removal of N-terminal proline residues from peptides [63, 64]. The biological role of this activity is not very well understood but may play a role in protein breakdown and recycling of amino acids. PhosPhAt predicts with a moderate confidence level that the PIP At2g14260 is phosphorylated at Thr137 within a minimal CDK consensus site. Similarly, the confirmed substrate IMD1, one out of three genes encoding 3-isopropylmalate dehydrogenases (E.C. 1.1.1.85), is also a predicted phospho-protein with high confidence for the minimal CDK phosphorylation sites and a medium confidence for the long CDK phosphorylation site (Table 2). Isopropylmalate dehydrogenases play a role in the leucine and glucosinolate biosynthesis pathways [65, 66]. Although glucosinolates are discussed as potential anticancer drugs, a direct link to cell growth and proliferation is not clear yet [67, 68].

The last substrate identified here is ALDEHYDE DEHYDROGENASE (ALDH), and both minimal CDK consensus sites are predicted in PhosPhAt to be phosphorylated (Thr193 [with high confidence] and Thr344 [with moderate confidence]). Recent evidence suggests that enhanced activity of specific ALDH aldehyde isoforms is a hallmark of cancer stem cells [69].

## Conclusion

We present here a pilot study that shows that analog-sensitive CDKs can be used in vivo to identify kinase substrates in a multicellular organism. This sets the base for future, more detailed and development-specific substrate searches. Interestingly, the here-identified substrates hint at many ways of how cell-cycle activity can be connected with other cellular functions. All of these substrates appear to be plant-specific substrates suggesting a largely species- or clade-specific way of integrating the cell cycle with development and physiology.

## Methods

### Modeling

The identity among CDKA;1 and its closest two human homologies Cdk1 and Cdk2 is about 66.6 %, indicating the high conservation of all three proteins. Structural data is only available from Cdk2. Therefore, a 3D model of CDKA;1 was generated using different templates of crystal structure data on human Cdk2. The modeling requests were submitted to SWISS-MODEL (http://swissmodel.expasy.org), a server for automated comparative modeling of three-dimensional protein structures [70]. For modeling in Fig. 1b, a structure of non-phosphorylated Cdk2 complexed with ATP and Mg^2+^ at 2.0 Å was chosen (PDB ID: 1B38) [71]. To model the structural changes of the CDKA;1-F80G point mutation shown in Fig. 1b, the CDKA;1 sequence was used for NCBI-BLAST searches against PDB with default settings delivering five hits with the same scores and E-values (score = 419 E-value = 7e-118); PDB ID: 2W17 [72], 1FIN [73], 3EZR [74], 3PXF [75] and 1GZ8 [76]. We decided to use 1FIN as the modeling template since the other structures showed several disordered regions with missing side chains atoms or missing residues. 1FIN is complete and contains coordinates of a bound ATP in the active site pocket. The alignment of CDKA;1 and the template sequence 1FIN was performed using ClustalX with default values. Modeller [77] was then used to compute the model of CDKA;1 with ATP as rigid body. All 3D views were prepared using PyMOL [78].

### Plant material and growth conditions

*Arabidopsis thaliana* (L.) Heyhn. seedlings, Columbia-0 ecotype (Col-0), were grown in a growth chamber on soil at 21 °C or on agar plates (0.75 % (w/v) agar containing half-strength Murashige and Skoog salt mixture (MS salt mixture, Sigma) and 1 % (w/v) sucrose (Santa Cruz Biotechnology, sc-204311). The *cdka;1-1* allele (SALK ID: 106809; in Col-0 background; obtained from the European Arabidopsis Stock Centre NASC, http://arabidopsis.info) was previously isolated [37] and used throughout this analysis as the reference allele, referred to as *cdka;1*. The PP1 analogs (4-amino-1-*tert*-butyl-3-(1′-naphthyl)pyrazolo[3,4-*d*]pyrimidine (1-NA-PP1) and 1-NM-PP1; Toronto Research Chemicals Inc.) were prepared as 10 mM stock solution in DMSO. To measure the root length, plants were grown vertically on agar plates (1 % (w/v) agar containing half-strength MS salt mixture and 1 % (w/v) sucrose supplemented with 10 μM 1-NM-PP1 or 0.1 % (v/v) DMSO). Selective media for establishing standard lines of *cdka;1-as* contained 10 mg/L DL-phosphinotricin (Santa Cruz Biotechnology, sc-263102). The plates were scanned with PERFECTION V750 PRO (Epson) and the root length was measured with ImageJ Simple Neurite Tracer plugin (http://fiji.sc/Simple_Neurite_Tracer).

### Cloning

All primers used in this study are listed in Additional file 2: Table S1. The *CDKA;1*^*F80G*^ pocket mutant variant was generated by fusion PCR with Pfu polymerase (Fermentas) from wild-type *Arabidopsis* CDKA;1 cDNA (gift of Dr. Christina Weinl, carrying a silent C180T point mutation) as a template. The primer combinations were ND10-ss_attB1:CDKcoreN and N275-Pocketas for the 5′ and N274-Pocketss and ND11-as_CDKcoreC:attB2 for the 3′ fragment, respectively. The two parts were cleaned with ExoSap-IT (USB) and fused in a final PCR with ND10-ss_attB1:CDKcoreN and ND11-as_CDKcoreC:attB2. The fusion was flanked by Gateway attB1 and −2 sites and recombined in pDONR201 (Invitrogen). The F80G substitution was achieved by changing T^238^T^239^t/Phe to G^238^G^239^t/Gly. To allow identification of the correctly altered CDKA;1 variant also later on from plant material, two silent restriction sites were introduced: silent G^234^>A for *Bfa*I and silent T^249^>A for *Xba*I. After sequencing, the obtained Gateway entry clones were recombined with a binary Gateway destination vector pAM-PAT-GW-ProCDKA;1 [37]. Resulting expression vectors conferring phosphinothricin resistance in plants were retransformed into *Agrobacterium tumefaciens* GV3101-pMP90RK [79] and transformed into heterozygous *cdka;1* mutants [37] by floral dip. The identity of the *Agrobacterium* strains was verified by back-transformation of isolated plasmid into *E. coli* and analytical digest [80].

To clone *CYCD2;1*, total RNA was extracted from a-week-old seedlings by using NucleoSpin RNA plant (Macherey-Nagel). First-stranded cDNA was synthesized by SuperScript III reverse transcriptase (Invitrogen) with oligo dT-AP_M13 according to the manufacturer’s instruction. *CYCD2;1* cDNA was amplified first with primers CYCD2;1_s1 and M13-forward, followed by primers CYCD2;1_s2 and CYCD2;1_RT with Phusion DNA polymerase (Thermo Scientific). The PCR product was cloned, by Gateway, into the pDONR201 vector, following by the amplification with a primer set attB1-CYCD2;1_s and attB2-CYCD2;1_as. A recombination reaction was performed between the resulting entry clone and a destination vector pHMGWA [81] by using LR Clonase II (Invitrogen).

cDNA clones of the substrate candidates were ordered from ABRC; FBA2 (U67655, At4g38970), IMD1 (S69273, At5g14200), PIP (U16854, At2g14260) mMDH1 (U16556, At1g53240.1) ALDH7B4 (U12536, At1g54100.1) pfkB-like (U18033, At2g31390.1). After they were subcloned, by Gateway, into pDONR223 (Invitrogen), sequences were confirmed. A recombination reaction was performed between the resulting entry clone and a destination vector pHGGWA [81].

### 2D-DIGE

Inflorescences of *cdka;1-as* (F80G) plants in *cdka;1*^*−/−*^ background were collected and frozen in liquid N_2_ then ground by using TissuLyser II (Qiagen). The resulting fine powder was thawed and suspended in IP buffer (25 mM Tris-HCl, 75 mM NaCl, 15 mM MgCl_2_, 15 mM EGTA, 0.1 % (w/v) NP-40, pH 7.5) containing protease inhibitors (Complete, EDTA-free; Roche), 1 mM NaF, 1 mM ß-glycerophosphate and 1 mM Na_3_VO_4_. Cell debris was pelleted by centrifugation at 20,000 × g, 4 °C, for 10 min, then the supernatant was again clarified by centrifugation at 20,000 × g, 4 °C, for 20 min. Buffer was exchanged with PD-Mini Trap G-25 column (GE Healthcare) to kinase buffer (50 mM Tris-HCl, pH 7.5, 10 mM MgCl_2_, 1 mM EGTA) containing protease and phosphatase inhibitors. Protein concentration was measured with a Bradford kit (Bio-Rad) by using BSA as a standard. 500 μg of total protein extracts were used in the kinase reaction in a total volume of 200 μl of kinase buffer containing 1 mM N^6^-(2-phenylethyl)adenosine-5′-O-(3-thiotriphosphate) (N6-PhEt-ATP-γ-S, Biolog) as a phosphate donor, and incubated for 4.5 h at 30 °C, then 2 μl of Nuclease Mix (GE Healthcare) were added and incubated for additional 30 min. After the kinase reaction, proteins were precipitated by adding 1.3 ml of ice-cold acetone and incubated at −20 °C overnight. After centrifugation of the tubes for 10 min at 12,000 × g at 4 °C, the pellet was washed twice with 1 ml 80 % acetone. Following another centrifugation step of 5 min at 12,000 × g at 4 °C, the supernatant was removed and the pellet was air-dried on the bench top. The pellet was re-suspended in 100 μl of IEF buffer (7 M urea, 2 M thiourea, 4 % (w/v) CHAPS, 20 mM Tris, pH 8.5) and protein concentration was determined using Bradford assay kit with BSA as the standard. For 2D-DIGE, the proteins were labeled with CyDye DIGE Fluors (minimal dyes, GE Healthcare) according to the manufacturer’s instructions. Briefly, 200 μg of proteins after the kinase reaction with N6-PhEt-ATP-γ-S were labeled with 400 pmol of Cy3 dye, and proteins after the kinase reaction without bulky ATP were labeled with 400 pmol Cy5 dye on ice for 30 min, in the dark. The labeling reaction was quenched with 0.2 mM lysine (Sigma). Following the labeling reaction, both reactions were mixed. After addition of DTT (Sigma) to a final concentration of 20 mM and ampholyte (Bio-Rad) to a final concentration of 0.2 %, as well as supplementation with bromophenol blue, the samples were applied onto immobilized pH gradient (IPG) strips (4.7–5.9 pH range, NL, 17 cm and 5–8 pH range, 24 cm, Bio-Rad). The strips were rehydrated with an IEF Cell apparatus (Bio-Rad) for 24 h, and subjected to isoelectrofocusing at 20 °C with limited amperage of 50 μA per strip as follows: after an active rehydration at 50 V, steps at 250 V and 6000 V were run for 15 min and 5 h, respectively. Voltage was then increased to 6000 V and IEF was stopped when 80,000 Vh were reached. The IPG strips were equilibrated for 15 min with gentle shaking in 375 mM Tris-HCl, pH 8.8, containing 6 M urea, 2 % (w/v) SDS, 2 %(w/v) DTT, 20 % (v/v) glycerol, and a trace of bromophenol blue. Iodoacetamide (Sigma, final concentration: 2.5 %(w/v)) was added to the second equilibration solution instead of DTT, and the strips were then incubated for 20 min in this solution. For the second dimension electrophoresis, 12 % SDS-PAGE gels were used at 25 mA per gel for 5 h. The fluorescent images were obtained with Ettan DIGE Imager (GE Healthcare) according to the manufacturer’s instructions. For the Col-0 samples, the proteins were applied onto IPG strips (3–10 pH range, 7 cm, Bio-Rad). The strips were rehydrated by using Ettan IPGphor 3 apparatus (GE Healthcare) for 12 h, and subjected to IEF at 20 °C with limited amperage of 50 μA per strip as follows: after an active rehydration at 50 V, the voltage was then increased to 4000 V and IEF was stopped when 10,000 Vh were reached. Prior to the second dimension, each gel strip was equilibrated as above, then proteins were separated on a 12 % Mini-PROTEAN TGX gel (Bio-Rad). The fluorescence images were obtained by Typhoon 9400 (GE Healthcare) at the Support Unit for Bio-Material Analysis in RIKEN BSI Research Resources Center (RRC).

### Mass spectrometric protein identification

After electrophoresis, gels were stained with colloidal Coomassie Brilliant Blue G-250, and scanned with GS-800 calibrated densitometer (Bio-Rad). Obtained gel images were analyzed with PDQuest 2-D Analysis Software (v. 8.0, Bio-Rad), and spots were picked with Robot Spot Cutter (Exquest, Bio-Rad). The gel digestion and subsequent MALDI-TOF/TOF measurements were carried out as described previously [82]. Proteins were identified by searching against the NCBI *Arabidopsis* protein database. Functional sites in candidate proteins were identified by using the ELM resource (http://elm.eu.org).

### Protein expression and purification

CDKA;1-CYCD2;1 complexes were expressed and purified by using a system as described previously [36]. After adding ATP at 2 mM, CDKA;1-CYCD2;1 complexes were incubated for 1 h at 30 °C. The reaction was then further purified with a column packed with Strep-Tactin sepharose (IBA), which had been equilibrated with kinase buffer. CDKA;1-CYCD2;1 complexes were eluted with kinase buffer containing 2.5 mM desthiobiotin. The aliquoted complexes were frozen in the liquid nitrogen and stored at −80 °C until use.

To express His:GST-fused proteins, *E. coli* BL21-AI cells (Invitrogen) for IMD1 and PIP, SoluBL21 cells (AMS Biotechnology) for DXR, FBA2, mMDH1, ALDH7B4 and pfkB-like, respectively, were transformed with the resulting vector. *E. coli* cells were grown in LB medium containing 100 mg/l ampicillin at 37 °C until OD600 = 0.6 and the production of the fusion protein was induced by adding 0.3 mM IPTG (and 0.2 %(w/v) L-arabinose (Sigma), in the case of BL21-AI cells) overnight at 18 °C. Cells were harvested by centrifugation and re-suspended in phosphate-buffered saline (PBS) buffer (140 mM NaCl, 2.7 mM KCl, 10.1 mM Na_2_HPO_4_, 1.8 mM KH_2_PO_4_, pH 7.3) containing the protease inhibitor (Complete; Roche), and lysed by sonication (Vibra-Cell, Sonics & materials). After addition of Triton X-100 to 0.2 %(w/v), the cell slurry was incubated at 4 °C and clarified by centrifugation. The supernatant was passed through a column packed with Glutathione-agarose (Sigma), which was washed sequentially with PBS, and eluted with Ni-NTA binding buffer (50 mM NaH_2_PO_4_, 100 mM NaCl, 10 %(v/v) glycerol, 25 mM imidazole, pH 8.0) containing 10 mM Glutathione. The eluate was sequentially purified with a column packed with Ni-NTA resin (Qiagen), which had been equilibrated with Ni-NTA binding buffer. His:GST-fused proteins were eluted with Ni-NTA elution buffer (Ni-NTA binding buffer containing 200 mM imidazole) and the buffer was exchanged to kinase buffer (50 mM Tris-HCl, pH 7.5, 10 mM MgCl_2_, 1 mM EGTA) with a PD-10 column (GE Healthcare). The concentration of proteins was adjusted to 0.5 mg/ml by using BSA as a standard.

### Protein blotting

Proteins were extracted from the inflorescences as described above. After protein extracts were quantified using the Bradford assay kit and 30 μg of total protein from each sample was separated on a 12.5 % SDS-PAGE gel (we use the acrylamide/bis solution (37.5:1, 2.6 % C), Carl Roth), proteins were transferred onto a PVDF membrane in the Towbin buffer with a wet blotting system (Bio-Rad), the membrane was then blocked with 5 %(w/v) non-fat dry milk in TBST (20 mM Tris-Cl, pH7.6, 137 mM NaCl, 0.1 %(v/v) Tween 20). To detect CDKA;1 proteins, the membrane was probed with a 1:5000 dilution of anti-PSTAIR monoclonal antibody (Sigma) and 1:10,000 HRP-conjugated anti-mouse antibody (KPL) in TBST. Enhanced chemiluminescence detection was performed with HRP substrate (Millipore). The signals were obtained by exposing a X-ray film.

### Kinase reaction

[γ-^32^P]-N6-PhEt-ATP was produced enzymatically by nucleoside diphosphate kinase (Sigma) in a procedure similar to a previously described method to generate bulky ATP versions [83]. The kinase assays were carried out with equal amounts of kinases, 2.5 μg of bovine histone H1 (Millipore) as a substrate, and 9.25 kBq of [γ-^32^P]-ATP (Hartmann Analytic) or [γ-^32^P]-N6-PhEt-ATP per reaction as previously described [36]. p13^Suc1^-associated kinases were purified from inflorescences of each genotype grown on soil and used in kinase assays as previously described [35], using histone H1 as a substrate. Kinase assays with recombinant CDKA;1-CYCD2;1 were performed as presented [36], using 2 μg of recombinant purified putative substrates.

To thiophosphorylate the substrate, GST-RBR1-His_6_ proteins were prepared as previously described [36]. The kinase reactions were carried out with equal amounts of kinases, 2 μg of GST-RBR1-His_6_ as a substrate, and 1 mM N6-PhEt-ATP-γ-S per reaction in kinase buffer for 30 min at 30 °C. After addition of p-nitrobenzyl mesylate (PNBM, Epitomics) to 2.5 mM, the reactions were further incubated for 1 h at 30 °C. The reaction was stopped by adding Laemmli sample buffer (Bio-Rad) then incubated for 3 min at 95 °C. Samples were separated on a 7.5 % Mini-PROTEAN TGX gel (Bio-Rad) and transferred with the Trans-Blot Turbo system (Bio-rad) according to the manufacturer’s instructions. The membrane was blocked with 5 %(w/v) skim milk (Wako Pure Chemical) in TBST (Bio-Rad). To detect thiophosphorylated GST-RBR1-His_6_ proteins, the membrane was probed with a 1:20,000 dilution of anti-thiophosphate ester rabbit monoclonal antibody (Epitomics) and 1:100,000 HRP-conjugated anti-rabbit antibody (GE Healthcare) in 5 % (w/v) skim milk in TBST. Enhanced chemiluminescent detection was performed with Clarity western ECL substrate (Bio-Rad). The images were obtained with LAS4030 Imager (GE Healthcare) according to the manufacturer’s instructions. After the blots were stripped with Restore PLUS stripping buffer (Thermo Scientific), the membrane was re-blocked with 5 % (w/v) skim milk in TBST and reprobed with a 1:20,000 dilution of HRP-conjugated anti-GST antibody (GE Healthcare).

### Identification of phosphorylation sites

To identify phosphorylation sites on IMD1 and mMDH1, kinase reactions were carried out with CDKA;1-CYCD2;1, 2 μg HisGST-IMD1 or HisGST-mMDH1, 1 mM ATP (Sigma) per reaction in kinase buffer with a final volume of 20 μl. After incubation for 1 h at 30 °C, the reactions were stopped by adding Laemmli sample buffer (Bio-rad) and boiled. Samples were separated on a 10 % TGX gel, and the gel were stained with Bio-Safe™ Coomassie G-250 Stain. An LTQ-Orbitrap XL (Thermo Fisher Scientific) coupled with an EASY-nLC 1000 (Thermo Fisher Scientific) was used for nano-LC-MS/MS analyses as described previously [84]. Raw data were processed using MaxQuant software (version 1.5.2.8, http://www.maxquant.org/) [85]. MS/MS spectra were searched by the Andromeda search engine against the *Arabidopsis* TAIR10_pep_20101214 database (ftp://ftp.arabidopsis.org/home/tair/Proteins/TAIR10_protein_lists/) and the sequences of His-GST-IMD1 and His-GST-mMDH1. Sequences of 248 common contaminant proteins and decoy sequences were automatically added during the search. Trypsin specificity was required and a maximum of two missed cleavages allowed. Minimal peptide length was set to seven amino acids. Carbamidomethylation of cysteine residues was set as fixed, oxidation of methionine, protein N-terminal acetylation and phosphorylation of serine, threonine, and tyrosine as variable modifications. Mass errors allowed were 20 ppm for peptides in the first search, 4.5 ppm in the main search and 0.5 Da for ion trap MS/MS fragment spectra. Peptide-spectrum-matches and proteins were retained if they were below a false discovery rate of 1 %. Extracted ion chromatograms (XICs) were calculated from the raw data in Xcalibur (version 3.1.66.10) allowing 10 ppm around the calculated peptide mass. MS1 precursor envelopes were created in Xcalibur by summing all spectra above 50 % of the maximum XIC intensity.

## Additional files

Additional file 1: Figure S1. Alignment of Cdk1-type kinases. A. Multiple alignment (MUSCLE) of Cdk1 (*H. sapiens*), Cdk2 (*H. sapiens*), Cdk3 (*H. sapiens*), CDC28p (*S. cerevisiae*), Cdc2^+^ (*S. pombe*) and CDKA;1 (*A. thaliana*). The amino acid modified in the gatekeeper mutant (F80 in CDKA;1) is marked by a star. B. Overlay of the computed 3D model of *Arabidopsis* CDKA;1 (turquoise ribbon) and the experimentally achieved X-ray structure of human Cdk2 (gray cartoon; PDB ID: 1B38) underscores the high conservation at the structural level of both kinases. The lack of gray ribbon on the lower left hand is due to a not resolved part of the structure in the template file. Given that HsCdk2 is a very good model for *Arabidopsis* CDKA;1, it can be used to further monitor the changes after mutation in its plant homologue as shown in Fig. 1b. Homology model built using SWISS-MODEL and graphics output generated using PyMOL (www.pymol.org). (JPG 2215 kb) Additional file 2: Table S1. Primer sequences used in this study. (XLSX 28 kb)

## Abbreviations

1-NA-PP1: 4-amino-1-*tert*-butyl-3-(1′-naphthyl)pyrazolo[3,4-*d*]pyrimidine
1-NM-PP1: 4-amino-1-*tert*-butyl-3-(1′-naphthylmethyl)pyrazolo[3,4-*d*]pyrimidine
6-Bn-ATP: N^6^-benzyladenosine-5′-O-triphosphate
2D-DIGE: Two-dimensional differential gel electrophoresis
ADK: Adenosine kinases
ALDH: ALDEHYDE DEHYDROGENASE
Cdc2/CDC28: Cell division cycle 2/28
Cdk1: Cyclin-dependent kinase 1
Cdk2: Cyclin-dependent kinase 2
CDKA;1: CYCLIN-DEPENDENT KINASE A;1
Cdka;1-as plant: Analog sensitive cdka;1 mutant with the following genotype: cdka;1 PRO_CDKA;1_:CDKA;1^F80G^
CYCD2;1: CYCLIN D2;1
IMD1: 3-isopropylmalate dehydrogenases
mMDH1: Mitochrondrial malate dehydrogenase
N6-PhEt-ATP: N^6^-(2-phenylethyl)adenosine-5′-O-triphosphate
N6-PhEt-ATP-γ-S: N^6^-(2-phenylethyl)adenosine-5′-O-(3-thiotriphosphate)
PIP: Proline iminopeptidase
PNBM: p-nitrobenzyl mesylate
PP1: 4-amino-1-*tert*-butyl-3-phenylpyrazolo[3,4-*d*]pyrimidine
