# Supplementary figures and images for: Modulation of plant growth in vivo and identification of kinase substrates using an analog-sensitive variant of CYCLIN-DEPENDENT KINASE A;1
Source: BMC Plant Biol. 2016 Sep 26;16:209. doi: 10.1186/s12870-016-0900-7 (PMC5037886; doi:10.1186/s12870-016-0900-7)

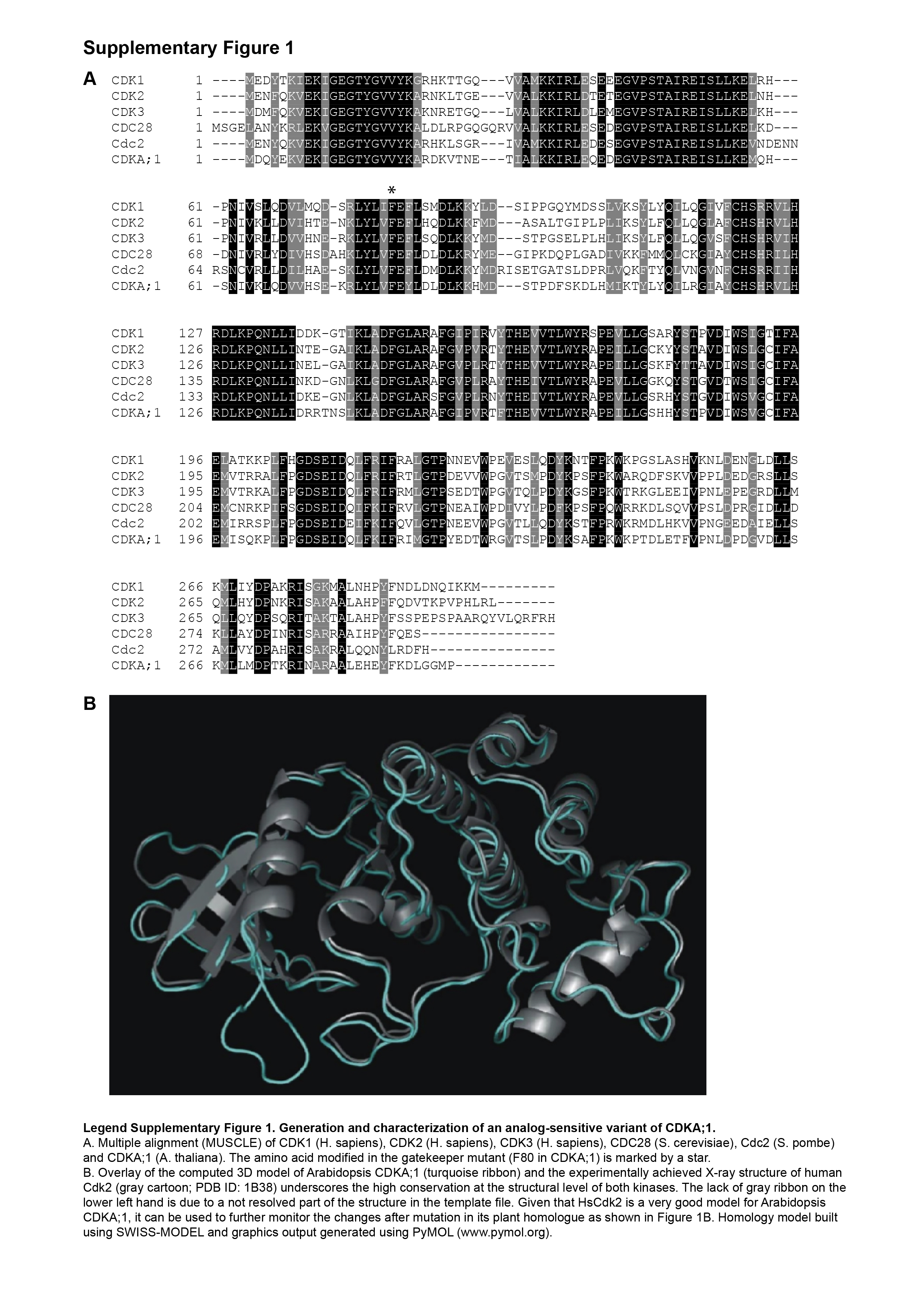

Supplement: Additional file 1: Figure S1. — Alignment of Cdk1-type kinases. A. Multiple alignment (MUSCLE) of Cdk1 (H. sapiens), Cdk2 (H. sapiens), Cdk3 (H. sapiens), CDC28p (S. cerevisiae), Cdc2+ (S. pombe) and CDKA;1 (A. thaliana). The amino acid modified in the gatekeeper mutant (F80 in CDKA;1) is marked by a star. B. Overlay of the computed 3D model of Arabidopsis CDKA;1 (turquoise ribbon) and the experimentally achieved X-ray structure of human Cdk2 (gray cartoon; PDB ID: 1B38) underscores the high conservation at the structural level of both kinases. The lack of gray ribbon on the lower left hand is due to a not resolved part of the structure in the template file. Given that HsCdk2 is a very good model for Arabidopsis CDKA;1, it can be used to further monitor the changes after mutation in its plant homologue as shown in Fig. 1b. Homology model built using SWISS-MODEL and graphics output generated using PyMOL (www.pymol.org). (JPG 2215 kb) [file 12870_2016_900_MOESM1_ESM.jpg]
